# Multiple Myeloma and lifetime occupation: results from the EPILYMPH study

**DOI:** 10.1186/1745-6673-7-25

**Published:** 2012-12-14

**Authors:** Carla Perrotta, Anthony Staines, Mary Codd, Silke Kleefeld, Dominique Crowley, Andrea T’ Mannetje, Nicholas Becker, Paul Brennan, Silvia De Sanjosé, Lenka Foretova, Marck Maynadié, Alexandra Nieters, Paolo Boffetta, Pierluggi Cocco

**Affiliations:** 1 Medicina Familiar y Comunitaria, Hospital Italiano de Buenos Aires, Buenos Aires, Argentina; 2 School of Nursing, Dublin College University, Dublin, Ireland; 3 School of Public Health and Population Sciences, University College Dublin, Dublin, Ireland; 4 Centre for Public Health Research, Massey University, Wellington, New Zealand; 5 German Cancer Research Center, Heidelberg, Germany; 6 International Agency for Research on Cancer, Lyon, France; 7 Catalan Institute of Oncology, CIBERESP, IDIBELL, Barcelona, Spain; 8 Department of Cancer Epidemiology and Genetics, Masaryk Memorial Cancer Institute, Brno, Czech Republic; 9 Dijon University Hospital, Dijon, France; 10 Centre of Chronic Immunodeficiency, University of Frieburg, Frieburg, Germany; 11 The Tisch Cancer Institute, Mount Sinai School of Medicine, New York, NY, USA; 12 International Prevention Research Institute, Lyon, France; 13 Department of Public Health, Occupational Health Section, University of Cagliari, Cagliari, Italy

**Keywords:** Multiple Myeloma, Occupation, Pesticide, Epidemiology, Case–control study, EPILYMPH study

## Abstract

**Background:**

The EPILYMPH study applied a detailed occupational exposure assessment approach to a large multi-centre case–control study conducted in six European countries. This paper analysed multiple myeloma (MM) risk associated with level of education, and lifetime occupational history and occupational exposures, based on the EPILYMPH data set.

**Methods:**

277 MM cases and four matched controls per each case were included. Controls were randomly selected, matching for age (+/− 5 years), centre and gender. Lifetime occupations and lifetime exposure to specific workplace agents was obtained through a detailed questionnaire. Local industrial hygienists assessed likelihood and intensity for specific exposures. The odds ratio and 95% confidence intervals (OR, 95% CI) were calculated for level of education, individual occupations and specific exposures. Unconditional logistic regression models were run for individual occupations and exposures.

**Results:**

A low level of education was associated with MM OR=1.68 (95% CI 1.02-2.76). An increased risk was observed for general farmers (OR=1.77; 95% CI 1.05-2.99) and cleaning workers (OR=1.69; 95% CI 1.04-2.72) adjusting for level of education. Risk was also elevated, although not significant, for printers (OR=2.06; 95% CI 0.97-4.34). Pesticide exposure over a period of ten years or more increased MM risk (OR=1.62; 95% CI 1.01-2.58).

**Conclusion:**

These results confirm an association of MM with farm work, and indicate its association with printing and cleaning. While prolonged exposure to pesticides seems to be a risk factor for MM, an excess risk associated with exposure to organic solvents could not be confirmed.

## Background

Multiple myeloma (MM) is a malignancy affecting differentiated B lymphocytes. It is uncommon in subjects under the age of fifty and the incidence rises sharply after the age of 65. It is more common in men, and among African Americans [1-3].

MM is a disease with a long latent period therefore exposures over extensive periods of time have interested researchers in particular agricultural occupations and exposure to pesticides [4,5]. Several studies observed an elevated MM risk in occupations with high exposure to organic solvents, such as painters, printers and petroleum workers [6,7].

The research into MM and occupation is extensive but most of the studies have failed to assess in detail occupational exposures and life-long occupational history. The complexity of measuring occupational exposure in a case–control setting—the most common study design study use to understand MM aetiology—is the primary reason for this.

Given this challenge, the EPILYMPH study applied a detailed occupational exposure assessment approach to a large multi-centre case–control study conducted in six European countries. Our goal in this paper is to assess MM risk associated with level of education, smoking, body mass index and lifetime occupational history and occupational exposures, based on the EPILYMPH data set.

## Methods

The EPILYMPH study is a multicentre case–control study on lymphoma aetiology conducted in 22 centres of six European countries (six centres in Germany, two in Italy, four in Spain, six in Ireland, three in France, and one in the Czech Republic). Case recruitment began in 1998 and ended in 2004. The methods are described in more detail in previous papers [8-10].

Controls were matched to cases in each centre by age (+/− 5 years) and gender. A random sample of the general population was selected to serve as a control in Germany and Italy. Meanwhile, centres in Ireland, France, Spain and the Czech Republic selected hospital controls, using uniform criteria for eligibility: patients were excluded as potential controls if the reason for hospitalization at the time of recruitment was cancer, organ transplantation, and/or a systemic infection.

Trained personnel conducted personal interviews for cases and controls alike using the same standardized questionnaire translated into the local language. The questionnaire addressed socio-demographic characteristics, including smoking status, weight and height, and -included a detailed lifelong occupational history.

Smoking status was categorized as ever smoker vs. never smoker, with those who smoked further analysed based on the number of packs per year. Weight and height were used to construct the Body Mass Index categorized using 30 as the cut-off point. Level of education was broken down into three categories: low level (primary school), medium level (high school) and high level (tertiary education).

The occupational history interview compiled a detailed lifelong job history, including job title, task description and duration. Based on the job title indicated, the participant was asked to provide additional detail using 15 special job modules which contained specific question on certain exposures—pesticides, organic solvents, living animals, working with children, radiation, dust—of prior interest to the researchers.

Each job was coded by a trained local industrial hygienist (IH) in participating countries. All job entries of one year or more listed under work histories were coded using the 1968 Standard Classification of Occupations (ISCO), and the 1990 Statistical Classification of Economic Activities in the European Community (NACE).

The ISCO coding system classifies jobs in a set of groups according to the tasks and duties undertaken in the job. ISCO codes can have a maximum of five digits. The first two digits describe broader occupational categories (e.g. 62 = general farmers). The third digit specifies the occupation in more detail (e.g. 621= ‘general farm workers’). The 4th and 5th digits further specify the task within the occupation group (e.g. 62200= ‘field crop and vegetable farm workers’). For the purpose of this study we used the more general first two digits of the codes, except for farming categories and exposure to organic solvents, for which the more detailed five-digit codes were used [11].

Duration of employment was categorized as <= 9 years and ≥ 10 years in reference to participants who never held that particular job title.

### Exposure

The EPILYMPH study investigated specifically the effect of several exposure groups: inorganic and organic pesticides, contact with live animals, dust, contact with meat, organic solvents, working with children and ionizing radiation.

The IH were trained to consistently assess exposure according to the job and task description. Three measures were used: frequency of exposure, intensity of exposure and confidence in the exposure assessment. Frequency of exposure was categorized as low (1) if the exposure occurred 1% to 5% of the working time; medium (2) if it occurred during 5% to 30% of working time; and high (3) if the exposure occurred more than 30% of the working time. Intensity of the exposure was categorized as low (1), medium (2) or high (3), with cut points for each agent based on the US Occupational Safety and Health Administration’s Threshold Limit Value (TLV), when available. Low intensity was defined as less than 50% of the TLV; medium was 50%-150% of the TLV, and high was more than 150% of the TLV. Intensity levels were based on a common standardized intensity scale with local and subject-specific estimates. When no TLV was available for a specific agent, benchmark occupations or tasks were used.

In terms of the third component, the IH expressed his/her own confidence in the exposure assessment using a three point scale, representing the degree of certainty that exposure to a specific substance had occurred (three being the highest). Duration of exposure was also recorded.

The study was approved by the Ethics Committee of each participating centre. All study subjects provided informed consent.

### Statistical analysis

A power calculation for this analysis was carried out to ascertain the minimum odds ratio (OR) to be detected with a 5% probability of α error and 80% of statistical power (ß=80), assuming a 20% prevalence of exposed subjects in the control group, a calculation based on the proportion of the control group who were farmers, resulting in a maximum matching ratio of controls to cases of 4:1.

Under such conditions, an OR of 1.7 would be detectable with 89% power. Consequently, four controls for each MM case were randomly selected from the total EPILYMPH controls, matching by age (+/− 5 years), gender and centre.

The OR and the respective 95% confidence interval were calculated using univariate analysis for level of education, smoking status, body mass index.

An unconditional regression model was used to deduct OR and 95% CI for each of the two-digit ISCO code categories adjusting for statistical significant co-variables.

The exposures were dichotomized into ever exposed /never exposed. For pesticide exposure and organic solvents, three additional variables were constructed: duration (no exposure, less than 10 years, 10 years or more); weighted cumulative exposure (duration x frequency), categorized in tertiles; and intensity categorized based on the IH classification as low, medium and high. A sub-group analysis by level of confidence was performed to test confidence-related changes in the risk estimates. The log-likelihood ratio was used to calculate the risk trend.

Sensitivity analysis was done to determine whether the risk varied between countries that selected population controls versus countries that selected hospital controls.

All the analyses were carried out using the Stata 9® software.

## Results

Complete occupational information was available for 277 of the 281 MM cases and the 1,108 controls randomly selected for analysis. The distribution of cases and controls by country and their demographic characteristics are shown in Table 1. The age distribution for both cases and controls was similar (mean age: cases 62.3, SD12.0; controls 62.4, SD 11.5); 57 % of the cases and controls were men, with a 1.3:1 male/female ratio.

**Table 1:** EPILYMPH study of multiple myeloma and lifetime occupation: demographic characteristics and geographical distribution of cases and controls

	**Cases**	**Controls**
Country (N, %)		
Czech Republic	32 (11.5 %)	128 (11.5%)
France	43 (15.5 %)	172 (15.5%)
Germany	75 (27.0 %)	300 (27.0%)
Ireland	27 (9.7 %)	108 (9.7%)
Italy	16 (5.7 %)	64 (5.7%)
Spain	84 (30.3 %)	336 (30.3%)
Total	277 (100%)	1,108 (100%)
Gender (N, %)		
Males	158 (57.0%)	632 (57.0%)
Females	119 (43.0%)	476 (43.0%)
Age (Years), Mean (SD)	62.3 (12.05)	62.4 (11.50)

A low education level prevailed among the cases (53.2%), compared to controls (49.7%) (Table 2); MM risk was elevated for low (OR=1.68, 95%CI 1.02-2.76) and medium education levels (OR=1.60, 95% CI 0.97- 2.65) compared to a high level. There was no excess risk associated with ever smoking or pack-year indicators, after adjusting for the other demographics covariates. Obesity, defined as BMI >=30, did not show an association with MM risk (Table 2).

**Table 2:** EPILYMPH Study of multiple myeloma and lifelong occupation: risk associated with selected covariates

	**Cases**	**Control**	**Adjusted OR (95% CI)**
	**(N=277)**	**(N=1,108)**	
Education, N (%)			
Low	147 (53.2%)	551 (49.7%)	1.68 (1.02 - 2.76)
Medium	108 (38.9%)	420 (37.9%)	1.60 (0.97 - 2.65)
High	22 (7.9%)	137 (12.3%)	1.00
Ever/never smoked, N (%)	150 (54.3%)	554 (55.4%)	0.96 (0.75 - 1.24)
Pack years, mean (SD)	25.7 (22 %)	23.6 (24.6%)	0.99 (0.98 - 1.01)
Body mass index >=30, N (%)	82 (31.1%)	277 (25.2%)	1.19 (0.91 - 1.56)

Occupations, industrial groups and MM risk.

The occupational groups with an increased risk were farmers (OR=1.77; 95% CI 1.05-2.99), cleaning workers (OR=1.69; 95% CI 1.04-2.72) and telephone and radio operators (OR=6.17; 95% CI 1.7-22.18). Printing workers showed a twofold increase in risk but this was not quite statistical significance. (OR= 2.06; 95% CI 0.97- 4.34) (Table 3).

**Table 3:** EPILYMPH study: Multiple Myeloma and lifelong occupation: Adjusted Odds Ratios by level of education, age, gender and centre and 95% CI with two-digit ISCO codes

**Job title (ISCO code)**	**N**	**N**	**OR**	**95% CI**
	**cases**	**controls**		
Teachers (13)	16	57	1.67	0.87 - 3.21
Managers (21)	12	39	1.44	0.73 - 2.74
Clerical supervisors (30)	53	248	0.40	0.03 - 3.96
Typists, stenographers (32)	31	115	1.20	0.43 - 3.35
Bookkeepers (33)	27	98	1.12	0.71 - 1.77
Telephone and telegraph operators (38)	6	4	6.17	1.71 - 22.2
Office clerks (39)	19	116	0.62	0.37 - 1.03
Insurance, real estate and auctioneers (45)	27	105	0.98	0.63 - 1.54
Cooks, waiters, related services (53)	18	69	0.99	0.57 - 1.70
Maids and housekeeping service workers (54)	25	86	1.11	0.69 - 1.79
Cleaners and related workers (55)	27	63	1.69	1.04 - 2.72
Farmers (61)	22	51	1.77	1.05 - 2.99
Agriculture and husbandry workers (62)	50	205	0.92	0.64 - 1.31
Spinners, dyers and related workers (75)	12	52	0.87	0.46 - 1.67
Tailors, sewers and related workers (79)	17	79	0.81	0.47 - 1.41
Blacksmiths, toolmakers and machine operators (83)	23	81	1.10	0.67 - 1.79
Plumbers, welders, and structural metal preparers (87)	12	52	0.86	0.45 - 1.65
Printers and related workers (92)	11	21	2.06	0.97 - 4.34
Bricklayers and construction workers (95)	31	122	0.97	0.64 - 1.48
Material handling and equipment operators (97)	24	83	1.1	0.68 - 1.79
Transport equipment operators (98)	18	93	0.71	0.41 - 1.20

Analysis of work duration showed that working more than 10 years as a farmer increased the OR (OR 2.03; 95% CI 1.13-3.65); printing workers also showed an increased risk with more than 10 years on the job, although non-significant(OR 2.71; 95% CI 0.94-7.77) (Results not shown).

The analysis of the more detailed agricultural tasks using the five-digit ISCO codes was limited due to the reduced number of exposed study subjects (results not shown). Gardeners had a nearly statistically significant increased risk (OR = 3.26; 95% CI 0.98-11.62).

### Occupational exposure

There was no increase in MM risk in the groups ever exposed to living animals, organic solvents, benzene, or any of the other specific exposure assessed in the EPILYMPH study (Table 4). The intensity categories did not have differences in MM risk estimates.

**Table 4:** EPILYMPH study of Multiple Myeloma and lifelong occupation

	**Cases (N)**	**Controls (N)**	**OR (95%CI)**
All pesticides	37	110	1.35 (0.90 - 2.02)
All inorganic pesticides	15	46	1.27 (0.69 - 2.33)
All organic pesticides	26	84	1.22 (0.77 - 1.95)
Contact with live animals	48	173	1.08 (0.75 - 1.54)
Contact with meat	30	118	0.96 (0.62 - 1.48)
Organic dust	156	585	1.07 (0.80 - 1.43)
Organic Solvents	114	435	0.96 (0.73 - 1.26)
Benzene	50	182	1.10 (0.78 - 1.56)
Toluene	50	182	1.10 (0.78 - 1.56)
Gasoline/petrol	72	278	1.03 (0.75 - 1.40)
Ionizing radiation	121	156	1.04 ( 0.79 - 1.33)
Contact with children	18	259	0.74 (0.44 - 1.25)

Adjusted OR by level of education, gender, age and centre and 95% CI for occupational exposures.

When duration was analysed there was an increased risk in workers exposed to pesticides more than ten years risk (OR=1.62; 95% CI 1.01-2.58).

The highest tertile of weighted exposure (frequency * duration) to pesticides had a non-significant excess risk (OR= 1.82; 95% CI 0.92-3.57, p for trend =0.09).

The highest tertile of weighted exposure for all the others exposure assessed did not showed differences in risk between MM cases and controls.

A sensitivity analysis was done, looking at the effect of the confidence level, as assessed by the local hygienists. This showed that there was no difference when analyses were restricted to exposures assessed with high confidence, as compared to those assessed with lower confidence. There were no differences in the estimated risks between countries that used hospital controls and those that used population controls.

## Discussion

The results of the analysis of MM and occupational exposure in the EPILYMPH dataset confirm previous suggestions of an increased risk of MM among farmers and its link with prolonged pesticide exposure. Cleaning workers also have an increase risk in line with a previous meta-analysis (4) and a case control study in non-Hodgkin lymphoma in New Zealand [12]. Those working as printers, for 10 years or more, had a marginally increased risk for MM. There were relatively few cases for both cleaners and printers therefore those results should be taken with care. This analysis also shows an increased risk for telephone and radio operators, although the estimate has wide confidence intervals. A previous study published in 1994 showed increased risk for MM in telephone-operators technicians [13]. It is likely that different exposures are in place nowadays in this particular job task.

Many previous observational studies explored the potential association between MM and farmers. A meta-analysis published in 1997 [14], concluded that farmers were at higher risk for MM than non-farmers. The pooled result from this meta-analysis from has significant unexplained heterogeneity that may lead to biased conclusions.

In a more recent systematic review, 22 case control studies were pooled and sources of heterogeneity were analysed [5]. Level of education variable was highlighted as one of the potential sources of heterogeneity as many older studies did not adjust for this variable. In this study there is a persistent increase in MM risk for general farmers and low level of education after mutual adjustment.

Which of all of the potential agricultural exposures may be responsible for the excess risk seeing in farmers? Our results did showed an increased risk in cases exposed to pesticides for more than 10 years. There is previous research linking pesticides and lymphomas as well, and an analysis of the Spanish EPILYMPH data set revealed an association between lymphoma and agricultural exposure to arsenic pesticides [15,16]. There is also evidence from the Agricultural Health Study of increased monoclonal gammopathy of undetermined significance prevalence in pesticide applicators [17].

There are other factors that may contribute to the aetiology of MM in farmers; contact with living animals [18,19] and exposure to organic solvents are among them [6]. In this analysis of the Epilymph data set, there was no increased risk following contact with live animals or husbandry workers or farmers exposed to organic solvents although numbers were relatively small which limits our capacity to to draw definite conclusions.

Cleaning workers showed an increased MM risk in this study. Cleaning workers are exposed to cleaning products which may have organic solvents; they are also exposed to disinfectants, perfumes and organic and inorganic dust [20,21].

MM risk was elevated for lower and medium education levels compared to to the highest level, which was not accounted for by occupations such as farmers or cleaning workers. This may suggest that conditions related to a poor education may be independent risk factors for MM. Future studies are warranted to address whether other surrogates for childhood socio-economic status would confirm this finding.

Smoking and body mass index did not show any link with MM in our study. Previous meta-analyses of obesity and MM showed an increased risk for the higher BMI categories (≥30 or with increases in BMI of 5 kg/m^2^) [22,23]. However, as obesity is more prevalent in lower socio-economic groups, other conditions also related to low socio-economic status could be acting as confounding factors. In this study, as in many others, Body Mass Index was calculated from the weight and height recorded at the time of the interview. Even though the study only recruited patients recently diagnosed with MM, it is possible that some patients have –at the time of interview- a lighter weight than their historical weight. This is a common limitation in case–control studies of lymphoma. There are several limitations to these results. This study was conducted in a total of 22 centres in six European countries. Considerable diversity in the pattern of occupational exposure across countries and within countries was to be expected. The purpose of assessing likelihood and intensity of workplace exposures with the aid of occupational experts was to improve the reliability of exposure and while there was a substantial training program, there may have been differences in the exposure assessment.

The duration cut-off limits was chosen arbitrarily. Probably a more sensitive approach would have been to choose less than 5, 5–9 and more than 9 but we did not have enough numbers on each category. The cut-off intends to separate people with long probability of being exposed to the same substances over a significant period of time. This is also arbitrary, as it is unlikely that the person would have been exposed, at the same level, during all that time. Unfortunately, that is an intrinsic limitation of observational studies, which take a snapshot of events that happened over a long period of time.

Although this is one of the single largest epidemiological studies of MM published to date, it might still suffer from inadequate power to detect the origins of the observed increased risk in farmers, and to find significant relationship with other occupations, such as painters and printers.

In conclusion, this multicentre collaborative study of lifetime occupation and risk of MM has shown an increase risk in farmers, cleaning workers and in subjects with prolonged exposure to pesticides. Further collaborative efforts will need to be pursued to better understand the observed associations.

## Competing interests

The authors do not have competing interest to disclosure.

## Authors’ contributions

CP conducted data acquisition for Ireland, data analysis, data interpretation and leaded manuscript writing and. AS, AM, NB, PB, SS, LF, AN, PC, and PB worked in the EPILYMPH study design, data gathering, individual countries studies. MC, DC and SK have worked setting up the EPILYMPH study in Ireland and occupational coding in Ireland. PC has also contributed to data interpretation. All authors read and approved the final manuscript.
